# Effect of substrate properties and phosphorus supply on facilitating the uptake of rare earth elements (REE) in mixed culture cropping systems of *Hordeum vulgare*, *Lupinus albus* and *Lupinus angustifolius*

**DOI:** 10.1007/s11356-022-19775-x

**Published:** 2022-03-28

**Authors:** Nthati Monei, Michael Hitch, Juliane Heim, Olivier Pourret, Hermann Heilmeier, Oliver Wiche

**Affiliations:** 1 Institute of Geology, Tallinn University of Technology, Tallinn, Estonia; 2 Biology/Ecology Unit, Institute of Biosciences, Technische Universität Bergakademie Freiberg, Freiberg, Germany; 3 Western Australian School of Mines, Curtin University, Bentley, Australia; 4 Environmental Microbiology Group, Institute of Biosciences, Technische Universität Bergakademie Freiberg, Freiberg, Germany; 5 AGHYLE, UniLaSalle, Beauvais, France

**Keywords:** Intercropping, Rhizosphere, Rare earth elements, White lupin, Root exudates, Phytoextraction

## Abstract

This study presents how phosphate (P) availability and intercropping may influence the migration of rare earth elements (REEs) in legume–grass associations. In a replacement model, *Hordeum vulgare* was intercropped with 11% *Lupinus albus* and 11% *Lupinus angustifolius*. They were cultivated on two substrates, A (pH = 7.8) and B (pH = 6.6), and treated with 1.5 g P m^−2^ or 3 g P m^−2^. Simultaneously, a greenhouse experiment was conducted to quantify carboxylate release. There, one group of *L. albus* and *L. angustifolius* was supplied with either 200 µmol L^-1^ P or 20 µmol L^-1^ P. *L. albus* released higher amounts of carboxylates at low P supply than *L. angustifolius*, while *L. angustifolius* showed the opposite response. Plants cultivated on substrate B accumulated substantially higher amounts of nutrients and REE, compared to substrate A. Higher P supply did not influence the leaf and stem P concentrations of *H. vulgare*. Addition of P decreased REE accumulation in barley monocultures on alkaline soil A. However, when *H. vulgare* was cultivated in mixed culture with *L. angustifolius* on alkaline substrate A with high P supply, the accumulation of REE in *H. vulgare* significantly increased. Conversely, on acidic substrate B, intercropping with *L. albus* decreased REE accumulation in *H. vulgare*. Our findings suggest a predominant effect of soil properties on the soil–plant transfer of REEs. However, in plant communities and within a certain soil environment, interspecific root interactions determined by species-specific strategies related to P acquisition in concert with the plant’s nutrient supply impact REE fluxes between neighbouring plants.

**Supplementary Information:**

The online version contains supplementary material available at 10.1007/s11356-022-19775-x.

## Introduction

Carboxylates released by plant roots are an important strategy of plants to access sparingly available phosphorus and micronutrients such as Fe and Mn in soil (Cu et al. 2005). Particularly for P, Fe and Mn, the availability is limited by low solubility of the corresponding element-bearing minerals and interactions with other inorganic and organic soil phases. Specifically, in soils, Fe and Mn are present as Fe oxyhydroxides and Mn oxides, characterized by low solubility above a soil pH of 5. Thus, in alkaline soils, the availability of Fe and Mn is limited by their extremely low solubility of the respective oxides and oxyhydroxides (Suda and Makino 2016) whereas phosphate strongly interacts with calcium by the formation of hardly soluble Ca phosphates. Moreover, under acidic soil conditions and below a pH of 7, Fe, Mn and P often behave in a dual way showing steadily increasing solubility of Fe and Mn, whereas fixation/specific sorption of phosphate on Fe oxyhydroxides and aluminium hydroxides increases and leads to accumulation of P in acidic soils in sparingly plant available forms.

Plants adapted to these conditions and evolved towards a self-determined influence on the chemical features surrounding their roots to create an environment which allows nutrient acquisition over a wide range of soil (rhizosphere) conditions. Besides mutualistic interactions with bacteria and fungi and alteration of soil physical properties, the most important and commonly explored mechanisms include acidification of the rhizosphere by release of protons and release of element-chelating carbon compounds such as carboxylates (Lambers 2022). The ability for mobilizing P and micronutrients in the rhizosphere varies considerably in dependence on plant species, functional plant groups (Neumann et al. 2000; Lambers et al. 2013, 2015) or even genotypes in a certain species (Krasilnikoff et al. 2003). Forbs in general and legumes in particular are considered to be P-efficient due to a strong ability to acidify the rhizosphere and release large quantities of carboxylates under P and Fe deficiency (Lambers et al. 2013; Nobile et al. 2019), while grasses such as *Avena sativa* and *Hordeum vulgare* are described as P-inefficient (Wang et al. 2013; Faucon et al. 2015; Lambers et al. 2015). Some forbs develop specialized root structures with abundant root hairs (cluster roots) that release large quantities of carboxylates into the rhizosphere and exhibit a highly efficient P mining strategy of which P mining strategies in *Lupinus albus* and species from the family Proteaceae have been the most profoundly studied (Lambers 2022).

Although these processes related to plant nutrition are initially regulated by nutrient deficiency, both strategies must be generally considered non-element-specific with respect to the effects of the chemical processes in the rhizosphere. That means while nutrient deficiency triggers a shift in metabolism towards elevated proton and carboxylate release, the compounds released do not only attack nutrient-bearing soil phases but also alter solubility and mobility of non-essential elements in the soil. In addition to this, they influence their availability as it has been demonstrated for Cd, Pb and rare earth elements (REEs) (Wiche et al. 2016a). Among these elements, REEs are particularly interesting to study because they (i) are present in almost all soils at concentrations comparable to essential plant nutrients; (ii) share chemical similarities to essential nutrients, particularly Ca; (iii) interact with nutrient-bearing soil minerals (phosphates, Fe oxyhydroxides); but (iv) are still not essential to plants nor strongly toxic (Tyler 2004).

More specifically, the REEs comprise a group of 17 elements from the lanthanide series including lanthanum, yttrium (Y) and scandium (Sc) that are abundant in the Earth’s crust with concentrations that vary from 66 µg g^−1^ (Ce), 30 µg g^−1^ (La) and 28 µg g^−1^ (Nd) to 0.3 µg g^−1^ (Lu) (McLennan 2001; Kabata-Pendias 2010; Davranche et al. 2017). As a special feature in this group, all 16 REEs exhibit ionic radii similar to Ca^2+^; however, under most pedological relevant conditions, REEs form + 3 ions (Wyttenbach et al. 1998) which strongly interact with phosphate and other negatively charged soil constituents (Diatloff et al. 1993; Zhimang et al. 2000; Cao et al. 2001; Li et al. 2014). As an exception in this group, Eu and Ce may also occur in the divalent or tetravalent state (Davranche et al. 2017). There are slight but indisputable differences in ionic radii from light REEs (LREEs) to heavy REEs (HREEs), leading to differences in their absorption and complexation behaviour in soil (fractionation). Consequently, this might also influence their movement in soil–plant systems and availability to plants. Previous studies conducted followed the generic laboratory and field approach, where synthetic REEs were introduced to the cultivation area. In other approaches, the cultivated plants were left to grow under natural conditions without any anthropogenic modifications (Cunha et al. 2012). There is general consensus that rhizosphere processes related to plant nutrition not only affect the availability of nutrients but also of non-essential elements such as Pb, Cd (Wenzel 2009) and REEs since these elements can be mobilized through lowering of pH and presence of organic acids (Wiche et al. 2017a). Under field conditions, Wiche et al. (2016a, b) demonstrated that mixed cultures of P-inefficient grasses with P-efficient legumes increase the uptake of REEs in the grasses which was most likely due to mobilization of REEs in the rhizosphere of lupins and movement of the elements between intermingling root systems which suggested that not the physiological mechanisms of uptake are of relevance for the accumulation levels of REEs in *A. sativa* and *H. vulgare*.

In fact, it is generally assumed that uptake of REE^3+^ ions is mediated mainly, but not solely by Ca^2+^, Na^+^ and K^+^ channels (Han et al. 2005; Brioschi et al. 2013; Wiche et al. 2017b). Thus, it seems that lupins are able to attack REE-containing soil phases through the release of protons and the exudation of organic acid anions, which renders these elements available for the P-inefficient grasses (Wiche et al. 2016b).

In the present study, we conducted a mixed culture study under field conditions where we cultivated *H. vulgare* (barley), a P-inefficient cereal in the presence of 11% lupins using either *L. albus*, a cluster root-forming legume (white lupin), and *Lupinus angustifolius*, a non-cluster root-forming lupin (narrow leaf lupin). Each of these cultivation forms was set up on two different soils with different soil pH values and thus differences in plant-available nutrients and REEs. Additionally, on each soil, the plant stands received P fertilizer at a rate of 1.5 g P m^−1^ and 3 g P m^−1^, respectively, to elucidate effects of P supply and soil properties on REE accumulation in monocultured and mixed cultured barley plants. Moreover, in a greenhouse experiment, we characterized the root exudate composition of both lupins depending on P supply which will give a hint on the plant’s behaviour at different P levels in the field. In combination, this ecologically derived experimental approach allows to explore the effects of soil nutrient availability and species-specific rhizosphere properties on REE dynamics in legume–grass associations which is a relatively unstudied research topic hitherto. Knowing the dynamics of the interaction of lupins and P in the rhizosphere, we hypothesise, firstly, that there is an interaction between P supply and REE accumulation in the plants and, secondly, this pattern should depend on the initial availability of nutrients in the substrates determining the nutritional status of the plants and REE mobility in the substrate. Lastly, the effects should depend on the lupin species and, consequently, on the amount and composition of root exudates interacting with soil phases in the intermingling rhizospheres of barley and lupins.

## Material and methods

### Field experiment

The experiment was conducted at Bauer Umwelt Business, Hirschfeld (Saxony, Germany), in its off-site recycling and remediation centre. A basin of a total volume of 720 m^3^ was filled with two homogeneously sieved top soils both characterized as Luvisols. One half of the basin was filled with soil from a road construction location near Freital, Germany (hereafter referred to as substrate A). The second half was filled with topsoil from a mining-affected area in the vicinity of Freiberg, Germany (hereafter referred to as substrate B). Substrate A was a silty loam with a pH (H_2_O) value of 7.9. Substrate B was a silty loam with a pH (H_2_O) value of 6.8 (Table 1). A summary of plant-available macronutrient concentrations in the two substrates used for the experiment is shown in Table 1. The elements P, Mg and K were extracted by calcium acetate lactate (CAL) and measured with inductively coupled plasma mass spectrometry (ICP-MS). For analysis of mineral N, NO_3_^−^ and NH_4_^+^ were extracted from soil samples and photometrically determined according to Bolleter et al. (1961) and Hartley and Asai (1963). NH_4_^+^ acetate (pH 5) was used for the extraction of exchangeable Ca which was determined through ICP-MS. Total concentrations of REEs, P, Ca, Fe and Mn of the substrate and their concentrations in six operationally defined soil fractions as a result of a sequential extraction according to Wiche et al. (2017a) for soil samples prior to the experiment are shown in Table 2. Both substrates were characterized by similar organic matter contents (LOI), CEC and macronutrient concentrations of N, P, K and Mg (Table 1); however, soil A displayed a significantly higher pH value compared to soil B, indicating differences in element availability. Total concentrations of P, Ca and Fe were significantly higher on substrate A compared to substrate B (Table 2). Also, substrate A contained higher concentrations of P, Ca, Mn and Fe in labile element fractions, especially exchangeable (F1), acid-soluble (F2) and organically bound elements (F3) whereas soil B was characterized by an enrichment of these elements in F4 and F5 (P, Ca, Fe) and F3 (Fe, Mn). In contrast, there were no differences in total concentrations between soils regarding Mn and REEs. The REEs showed no difference in fraction 1 and fraction 2 but showed a substantial enrichment of LREEs in F3 of soil B, leading to a 24% higher labile LREE pool in soil B compared to soil A (Table 2).

**Table 1:** Characteristics of the two different substrates used in the semi-field experiment and initial nutrient concentrations at the beginning of the experiment

Sample	pH (H_2_O)	LOI%	CEC_eff_ (cmol kg^−1^)	N_min_ (mg kg^−1^) (dw)	P_CAL_	K	Mg
Soil A	7.9 ± 0.4	7.8 ± 1.2	15.6 ± 2.3	47 ± 17	23 ± 9	462 ± 137	243 ± 89
Soil B	6.8 ± 0.3	6.4 ± 1.3	14.0 ± 3.0	32 ± 9	34 ± 6	284 ± 66	170 ± 78

The values are means of 20 replicates for each soil (means ± SD)

*LOI* loss of ignition, *CEC*_*eff*_ effective cation exchange capacity, *N*_*min*_ mineral N, *P*_*CAL*_ calcium acetate/lactate extractable phosphate

**Table 2:** Total concentration and sequential extraction results (µg g^−1^ dw) for the identification of the total concentrations of trace elements in the soil substrates

Fraction	P	Ca	Mn	Fe	LREE	HREE	LREE/HREE
Substrate A							
Total	1009 ± 213a	12,292 ± 4595a	977 ± 280	31,087 ± 21,848a	109 ± 27	34 ± 7.7	3.2 ± 0.37
F1	31 ± 16a	4526 ± 1526a	77 ± 25a	3.52 ± 1.06a	0.3 ± 0.08	0.1 ± 0.03	0.3 ± 0.03
F2	57 ± 12	1078 ± 436a	194 ± 35a	222 ± 74.3	3.7 ± 0.7	1.4 ± 0.3	0.4 ± 0.02
F3	133 ± 164	409 ± 214a	112 ± 48b	780 ± 1033b	7.6 ± 5.0b	2.2 ± 0.2b	0.6 ± 0.7a
F4	1121 ± 400a	75 ± 24	42 ± 35	6508 ± 2231b	11 ± 4.5	2.5 ± 1.2	0.2 ± 0.04
F5	73 ± 23a	212 ± 80.1	29 ± 12	4756 ± 1203b	3.3 ± 0.8	0.8 ± 0.2a	0.3 ± 0.03
Substrate B							
Total	878 ± 236b	5775 ± 1619b	887 ± 250	25,296 ± 21,848b	106 ± 19	34 ± 6.6	3.1 ± 0.23
F1	20 ± 13b	2955 ± 882b	47 ± 12b	2.5 ± 0.8b	0.3 ± 0.05	0.09 ± 0.01	0.3 ± 0.03
F2	50 ± 21	513 ± 239b	118 ± 30b	181 ± 85.7	3.2 ± 0.7	1.0 ± 0.2	0.3 ± 0.02
F3	169 ± 135	243 ± 79.2b	198 ± 27a	1401 ± 930a	9.4 ± 3.2a	2.6 ± 0.8a	0.3 ± 0.15b
F4	1496 ± 412b	69 ± 20	38 ± 18	8049 ± 1777a	11 ± 4.0	2.3 ± 0.8	0.1 ± 0.01
F5	110 ± 21b	237 ± 84.7	31 ± 5.4	6396 ± 557a	3.0 ± 0.6	0.7 ± 0.1b	0.3 ± 0.02

Given are means ± SD (*n* = 10). Concentrations within the same element fraction between the substrates were compared by *t* tests with Bonferroni adjustment. Means with different letters are statistically significantly different at *α* = 5%

*F1* exchangeable elements, *F2* acid-soluble elements, *F3* elements in oxidizable matter, *F4* amorphous oxides, *F5* crystalline oxides (Wiche et al. 2017b)

### Plant cultivation

White lupin (*Lupinus albus* L. cv. Feodora), narrow leaf lupin (*Lupinus angustifolius* L. cv. Sonate) and barley (*Hordeum vulgare* L. cv. Modena) were grown within field conditions in both a monoculture and a mixed culture system on 50 plots with an area of 4 m^2^ each (Online Resource 1). To avoid interactions between adjacent plots (e.g. root interactions, water discharge, nutrients, REE, and trace metals), a 0.5-m buffer zone was maintained surrounding each plot without vegetation. On each of the experimental plots, the plants were planted in rows (leaving 20 cm between rows) with a total density of 400 seeds m^−2^. Mixed barley cultures were obtained from the monocultures by replacement of 11% barley plants with the equivalent proportion of individuals of white lupin and narrow leaf lupin, and thus plant densities were equivalent in both barley monocultures (hereinafter referred to as L0 plots) and mixed cultures (hereinafter referred to as Lal and Lan plots, respectively) (Online Resource 1).

Eight days after seed germination and plant development had taken place, the first dose of fertilizer was given to all plants. Each substrate plot with barley monocultures and mixed cultures with white and narrow leaf lupin (Lal and Lan) was dosed with 10 g of N m^−2^ as NH_4_NO_3_, 11.6 g of K m^−2^ as KNO_3_, 3 g of P m^−2^ as KH_2_PO_4_ and 1.5 g of Mg m^−2^ as MgSO_4_, representing the fully fertilized reference plants (NPK). Accordingly, each substrate plot of barley monocultures (L0) and mixed cultures with narrow leaf lupin (Lan) received a similar fertilizer composition regarding N, K and Mg but with one half of P (1.5 g of P m^−2^ as KH_2_PO_4_) representing the low-dosed variant (NK). To ensure consistency in the provision of nutrients throughout the entire experiment and to avert N deficiency (e.g. by leaching nitrate), the abovementioned fertilizer was applied in two separate doses at the beginning of the experiment and a second time 4 weeks later.

Each of the different treatments, including culture forms and fertilizer treatment, was fivefold replicated on each of the two substrates, and within each substrate, the treatments were set up in a fully randomized design. After 8 weeks of plant growth, shoots of barley in monocultures and mixed cultures were cut 3 cm above the soil surface when harvesting. Only the shoots of the inner square meter of each plot were further processed for chemical analysis.

### Quantification of carboxylate release

A separate greenhouse experiment was designed for the determination of root exudates in both *L. albus* and *L. angustifolius* depending on P supply. Seeds of *L. albus* cv. Feodora and *L. angustifolius* cv. Sonate were surface sterilized by washing the seeds with 0.5% sodium hypochlorite (NaOCl) for 3 min followed by carefully rinsing with deionized water and allowed to germinate in petri dishes in a climate chamber at 20 °C. After germination, the seedlings of each plant species (one seedling per pot) were planted in 10 plastic pots (2 L total volume) filled with acid (HNO_3_)-washed quartz sand. The pots were incubated in a greenhouse for 6 weeks with a 15-h photoperiod, temperature of 18–30 °C, relative humidity of 65% and average photosynthetically active photon flux density of 630 µmol m^−2^ s^−1^. During a time period of 4 weeks, all plants received weekly 200 mL of a modified nutrient solution according to Arnon and Stout (1939), supplying all necessary plant nutrients except phosphorus. Additionally, for each species, one-half of the plants received 200 µmol L^−1^ K_2_HPO_4_ together with the other nutrients (high P) while the other plants received 20 µmol L^−1^ P (low P references). After a cultivation period of 4 weeks, the mature plants were carefully removed from the sand by washing with tap water and transferred into glass beakers containing 300 mL of a 2.5 µmol L^−1^ CaCl_2_ solution where they were let to stay for 2 h under a growth lamp and allowed to release carboxylates into the collection solutions. Immediately after the collection, the resulting solutions were stabilized with 1 mL L^−1^ Micropur to prevent microbial decomposition of carboxylates according to Oburger et al. (2014) and analysed by means of ion chromatography. Thereafter, the shoots and roots were separated, weighed and dried for 24 h at 60 °C.

### Analysis of trace element concentrations and carboxylates

The harvested biomass of field grown plants was separated in leaves and stems and dried at 60 °C in an oven for 24 h. The dried biomass was ground to fine powder and stored in centrifuge tubes. Thereafter, microwave digestion (Ethos plus 2, MLS) was carried out with 0.1 g of the subsample taken from the ground biomass measured in duplicates. Samples were mixed with 1.6 mL nitric acid (65% supra) and 0.6 mL hydrofluoric acid (4.9% supra) and heated to 220 °C in the microwave according to Krachler et al. (2002). Concentrations of P, Fe, Mn, Ca, Mg and REEs (Y, La, Ce, Pr, Nd, Sm, Eu, Gd, Tb, Dy, Ho, Er, Tm, Yb and Lu) from the diluted digestion solutions and soil solutions were determined by ICP-MS (XSeries 2, Thermo Scientific) using 10 µg L^−1^ rhodium and rhenium as internal standards.

Concentrations of acetate, malonate, fumarate, glutarate, malate and citrate in the collection solutions were determined by ion chromatography equipped with suppressed conductivity detection (ICS-5000, 4-mm system, Thermo Scientific). Inorganic and organic acid anions were separated at 30 °C on an IonPac® AS11-HC column (Thermo Scientific) using gradient elution with sodium hydroxide as eluent and a flow rate of 1.0 mL min^−1^. The measuring program started with an 8-min isocratic phase and a sodium hydroxide concentration of 1 mmol L^−1^, followed by the gradient analysis with a continuously increasing sodium hydroxide concentration up to 40 mmol L^−1^ over a period of 35 min. Finally, the column was flushed for 3 min with 50 mmol L^−1^ sodium hydroxide and equilibrated for 10 min with 1 mmol L^−1^ sodium hydroxide.

### Data processing and statistical analysis

Concentrations of LREEs and HREEs in the plant and soil samples were calculated according to Tyler (2004) as the sum of La, Ce, Pr, Nd, Pm, Sm and Eu (LREEs) and Gd, Tb, Y, Ho, Er, Yb, Tm and Lu (HREEs). All element concentrations reported were calculated on dry weight basis. Significant differences among means of element concentrations in soil fractions, carboxylate concentrations of high-P- and low-P-treated plants and P concentrations in lupines cultivated with different P supplies were compared by *t* test with Bonferroni adjustment of *p* values using IBM SPSS Statistics 25. Additionally, concentrations and contents in different plant parts of the same plants were compared by a *t* test for non-independent samples at *α* = 5%. Means of plant yield, element concentrations and contents (calculated as concentrations × biomass) in different plant parts resulting from different culture forms (monocultures and mixed cultures with different lupins) as well as factors contributing to altered plant accumulation were evaluated by multifactor multivariate analysis of variance (MANOVA) using a type III model. In case of significant effects indicated by a significant Wilks’ lambda at *p* < 0.05, Duncan’s post hoc test was used. Prior to the analysis, the data was checked for homogeneity of variances using Levene’s test. In case that the assumption of homogeneity was violated, the data was log transformed. If the assumption was still violated, significant differences of means were identified by using single comparisons of groups of means using Welch’s ANOVA at *α* = 5%.

## Results

### Root exudate patterns in L. albus and L. angustifolius affected by P supply

Compared to *L. angustifolius*, *L. albus* produced higher shoot (high P [203%], low P [137%]) and root biomass (high P [400%], low P [233%]), irrespective of P supply (Table 3). P supply did not influence the root and shoot dry mass in *L. angustifolius* as well as the root dry mass in *L. albus*. However, the shoot dry mass of *L. albus* responded to differences in P supply showing a reduction by 35% when plants were supplied with low P doses. From the carboxylates measured, only citrate and malate were detectable in all collection solutions (Table 3), while fumarate was only occasionally present. All other carboxylate signals (acetate, lactate, glutarate, malonate) were below their respective detection limits. Under conditions of low P supply, *L. albus* strongly responded by 271% increased rates of citrate release per unit root dry mass and showed a 71% increased release of citrate per plant (Table 3). In this study, P supply did not alter the release of malate by *L. albus*. In contrast, in *L. angustifolius*, P deficiency did not increase the release of carboxylates. Instead, in *L. angustifolius* in adequately P-supplied plants, exudation rates of citrate and malate per unit root dry mass were 224% and 243%, respectively, higher than those in P-deficient plants. Overall, in *L. angustifolius*, this resulted in a 180% higher release of citrate and 650% higher release of malate in P-supplied plants. A comparison of exudation rates and amounts of carboxylate release per unit root dry mass between two lupin species revealed that there was no difference in the exudation rates under low P supply. However, when the plants received high P doses with the treatment solutions, exudation rates of citrate and malate in *L. angustifolius* per unit root dry mass were 1100% (citrate) and 140% (malate) higher than those in *L. albus* (*p* < 0.05). Considering the amounts of carboxylates released per plant individual (µM h^−1^) under P deficiency, *L. albus* released 140% and 900% more citrate and malate, respectively. In contrast, when P supply was high, *L. angustifolius* released 100% more citrate while the release of malate was similar.

**Table 3:** Growth parameters and root carboxylates collected from *L. albus* (Lal) and *L. angustifolius* (Lan) that were semi-hydroponically cultivated under P-deficient conditions (20 µM P: low P) or supply of 200 µM P (high P)

Species	P supply	Growth parameter		Release per plant			Release per dry weight		
		Root dw, g	Shoot dw, g	Citrate, µM h^−1^	Malate, µM h^−1^	Fumarate, µM h^−1^	Citrate, µmol (g dw h^−1^)^−1^	Malate, µmol (g dw h^−1^)^−1^	Fumarate, µmol (g dw h^−1^)^−1^
Lal	High P	0.8 ± 0.2	2.3 ± 0.4	0.7 ± 0.1	0.6 ± 0.4	0.02 ± 0.01	0.8 ± 0.1	1.0 ± 0.3	0.08 ± 0.07
	Low P	0.6 ± 0.3	1.5 ± 0.7	1.2 ± 0.1	0.8 ± 0.2	< 0.01	3.0 ± 1.4	1.1 ± 0.6	< 0.02
*p* value		0.43	0.08	< 0.01	0.24	0.34	0.03	0.91	NA
Lan	High P	0.16 ± 0.13	0.76 ± 0.45	1.4 ± 0.5	0.6 ± 0.3	< 0.01	9.4 ± 4.1	2.4 ± 0.6	< 0.06
	Low P	0.18 ± 0.08	0.59 ± 0.21	0.5 ± 0.3	0.08 ± 0.01	< 0.01	2.9 ± 0.4	0.7 ± 0.3	< 0.06
*p* value		0.88	0.82	0.06	0.04	NA	0.04	0.01	NA
*p* value	High P	< 0.01	< 0.01	0.04	0.83	NA	0.02	0.01	NA
	Low P	0.22	0.07	0.04	0.01	NA	0.95	0.43	NA

The values are means ± SD (*n* = 4). Significant differences among parameters within a species and between species and within a specific P treatment were identified by a *t* test with Bonferroni adjustment

*NA* not available

### Plant growth and nutrient concentrations in monocultured and mixed cultured barley plants

In all experimental units, biomass of *H. vulgare* shoots consisted mostly of stem biomass which, on average, yielded 122% more biomass per unit area than that of leaves (Table 5). Substrate properties, culture form (mixed culture with different mixing ratios of *L. albus* or *L. angustifolius*) and P fertilization did not influence the biomass yields of stems of *H. vulgare* (Tables 4 and 5), and there were no differences in leaf biomasses between substrates. Also, intercropping and P addition did not influence the leaf biomass on substrate B, neither in plant stands with *L. albus*, nor with *L. angustifolius*. However, on substrate A, mixed culture cultivation with *L. angustifolius* slightly increased (*p* = 0.09) the leaf biomass of barley when barley was cultivated at low P application level (NK) (Table 4) showing a 126% higher leaf biomass compared to the monocultures. This increase resulting from intercropping was not observable in NPK-treated plants on substrate A, and thus, leaf biomasses in mixed cultures grown under NK addition were by 195% higher (*p* = 0.06) compared to those in barley plants grown in NPK-treated mixed cultures.

**Table 4:** Multifactor multivariate ANOVA based on leaf and stem concentrations of barley plants exploring for effects of the growth substrate, fertilizer addition (3 g m^−2^ P or 1.5 g m^−2^ P, respectively) and culture form (monocultures and mixed cultures)

Plant tissue	Source of variation	Yield	P	Ca	Mn	Fe	LREE	HREE	L/H
Leaves	Substrate	NS	(*)	***	***	***	**	*	NS
	Fertilizer	(*)	NS	NS	NS	NS	NS	NS	NS
	Culture	*	NS	(*)	**	NS	NS	NS	NS
	Substrate × culture	NS	NS	NS	NS	NS	NS	NS	NS
	Fertilizer × culture	NS	NS	NS	NS	NS	*	*	(*)
Stems	Substrate	NS	**	*	**	NS	NS	NS	NS
	Fertilizer	NS	NA	NA	NS	NS	NS	NS	NS
	Culture	NS	NS	NS	NS	NS	(*)	NS	*
	Substrate × culture	NS	*	NS	NS	*	**	*	NS
	Fertilizer × culture	NS	NS	NS	NS	NS	NS	NS	NS

*NS* not significant

(*) *p* < 0.1; *p < 0.05; **p < 0.01; ***p < 0.001

**Table 5:** Yield of leaves and stems and concentrations of phosphorus (P), calcium (Ca), manganese (Mn) and iron (Fe) in the plant parts of *H. vulgare* depending on substrate (slightly alkaline substrate A and slightly acidic substrate B), P addition as fertilizer (NK: 1.5 g m^−2^ P; NPK: 3 g m^−2^ P) and culture form (monoculture: L0, mixed culture with 11% *L. albus* (Lal) and mixed culture with 11% *L. angustifolius* (Lan))

Culture form	Culture	Leaves					Stems				
		Yield, g m^−2^ dw	P, mg g^−1^ dw	Ca, mg g^−1^ dw	Mn, µg g^−1^ dw	Fe, µg g^−1^ dw	Yield, g m^−2^ dw	P, mg g^−1^ dw	Ca, mg g^−1^ dw	Mn, µg g^−1^ dw	Fe, µg g^−1^ dw
Substrate A											
Fertilizer											
NK	L0	85 ± 13b	1.9 ± 0.4*B*	5.6 ± 1.9	13 ± 3*B*	117 ± 36*B*	155 ± 46	1.4 ± 0.5(b)*B*	2.2 ± 0.5	4.5 ± 0.9*B*	25 ± 9*B*
	Lan	192 ± 116a(A)	2.3 ± 0.5	6.4 ± 1.3	16 ± 3*B*	146 ± 41*B*	317 ± 186	2.3 ± 0.6(a)	2.2 ± 0.2	5.7 ± 0.7*B*	28 ± 5
NPK	L0	65 ± 12	2.3 ± 0.4	5.2 ± 0.9(a)	12 ± 2(a)*A*	111 ± 15*A*	192 ± 41	1.3 ± 0.6*B*	2.1 ± 0.4	5.9 ± 3.2*B*	29 ± 7
	Lan	65 ± 17(B)	2.1 ± 0.4	6.9 ± 1.3(b)*A*	14 ± 2(a)*A*	152 ± 55B	153 ± 26	1.9 ± 0.5	2.3 ± 0.6	5.7 ± 0.8*B*	34 ± 6
	Lal	53 ± 12	2.2 ± 0.4	5.3 ± 0.9(a)	24 ± 7(b)	140 ± 32	157 ± 66	1.3 ± 0.7	2.1 ± 0.4	5.6 ± 1.2	41 ± 10
Substrate B											
Fertilizer											
NK	L0	92 ± 41	2.5 ± 0.8*A*	6.8 ± 1.8(b)	41 ± 4*A*	204 ± 66*A*	158 ± 41	2.5 ± 0.4*A*	2.4 ± 0.5	19 ± 4*A*	48 ± 8(A)*A*
	Lan	95 ± 40	2.0 ± 0.2	8.6 ± 1.2(a)	35 ± 13*A*	202 ± 17*A*	123 ± 23	2.7 ± 0.5	2.8 ± 0.8	12 ± 6*A*	36 ± 7
NPK	L0	61 ± 17	2.1 ± 0.6	8.9 ± 4.8	40 ± 23(a)*B*	177 ± 45*B*	164 ± 47	2.1 ± 0.6*A*	2.7 ± 1.2	15 ± 12*A*	28 ± 6(a)(B)
	Lan	77 ± 32	2.3 ± 0.4	9.3 ± 1.5*B*	47 ± 13(a)*B*	196 ± 61*A*	127 ± 65	2.3 ± 0.4	2.9 ± 0.4	17 ± 9*A*	44 ± 11(b)
	Lal	50 ± 14	2.4 ± 0.5	10.4 ± 3.5	101 ± 49b	184 ± 23	135 ± 34	2.2 ± 0.3	3.6 ± 0.8	16 ± 6	31 ± 6(a)

Means ± SD (*n* = 5). Significant differences in yields and concentrations within a plant part and substrate were identified by MANOVA followed by Duncan’s post hoc test. Small letters show differences between means of monocultured and mixed cultured barley within a specific substrate and P treatment. Capital letters denote differences of concentrations in barley plants of a specific treatment between P treatments within a substrate. Capital letters in italics show differences of concentrations in barley plants between substrates at *α* = 0.05

A comparison of concentrations in leaves and stems, respectively, and considering data from both substrates and all culture forms and fertilizer treatments revealed that concentrations of all investigated elements were consistently higher in leaves compared to the stems, except for P on substrate B. On substrate A, leaf concentrations were 28% (P), 171% (Ca), 196% (Mn) and 316% (Fe) higher than stem concentrations. On substrate B, leaf concentrations were 201% (Ca), 213% (Mn) and 405% (Fe) higher than stem concentrations.

Compared to the reference plants treated with 1.5 g P m^−2^, the addition of 3 g m^−2^ P did not affect the concentrations of Ca, Fe, Mn and P in leaves and stems, respectively, irrespective of the growth substrate. The growth substrate strongly affected concentrations of Ca, Mn and Fe (*p* < 0.01) and slightly affected P concentrations (*p* < 0.1) in leaves, while in stems, the growth substrate highly affected P, Ca and Mn concentrations with no significant effects on Fe. Specifically, considering all data from mixed culture types (*L. albus* and *L. angustifolius*) and P fertilizer treatments, leaf concentrations on substrate B were 13% (P), 45% (Ca), 213% (Mn) and 44% (Fe) higher than those on substrate A. In the same manner, stem concentrations of plants cultivated on substrate B were 43% (P), 31% (Ca) and 220% (Mn) higher than those on substrate A. Moreover, besides major effects of the substrate, multifactor MANOVA revealed significant effects of intercropping (culture form) on Mn in leaves (*p* < 0.001) and marginally significant effects on Ca (*p* = 0.08), while in the tillers, concentrations of P and Fe exhibited significant substrate–culture interactions, indicating that the effect of culture form depends on the growth substrate. More specifically in both substrates, concentrations of Ca increased by 33% and 26% in leaves of *H. vulgare* when the plants were cultivated in mixed cultures with *L. angustifolius* compared to the monocultures (L0), whereas there was no significant effect from *L. albus*. Additionally, leaf Mn concentrations increased highly significantly (*p* < 0.01) as an effect of mixed culture cropping with *L. albus* by 100% on substrate A and by 153% on substrate B, while the presence of *L. angustifolius* did not influence Mn in mixed cultured barley. In the stems, mixed cultures with *L. angustifolius* increased the P concentration significantly by 64% (*p* = 0.06) compared to the monocultures but this effect was only visible on substrate A. The presence of *L. angustifolius* significantly increased Fe concentrations in tillers of barley by 57%, but this effect was only observable on substrate B. Compared to the leaves, there was no effect of the mixed cultures on Ca and Mn in tillers of mixed cultured barley, and compared to *L. albus*, the presence of *L. angustifolius* led to more substantial changes in mineral element composition of *H. vulgare*, except for Mn which was highly affected by *L. albus*.

### Rare earth element concentrations in different plant parts

Considering both substrate types, all culture forms and fertilizer treatments, concentrations of REEs were constantly higher in leaves compared to those in stems with LREE/HREEs > 1 (Table 6). On substrate A, leaf concentrations were 442% (LREEs) and 140% (HREEs) higher than stem concentrations (*p* < 0.01). Also, the LREE/HREE ratio was 46% higher in leaves than in stems (*p* < 0.01). On substrate B, leaf concentrations were 540% (LREE) and 280% (HREE) higher in leaves than in stems (*p* < 0.01) with very similar LREE/HREE ratio among the two plant compartments. The addition of P fertilizer did not affect the concentrations of REEs directly (Tables 4 and 6). However, there were significant interaction effects between P application and culture form influencing the REE concentrations in the leaves as well as P application × culture interactions influencing the REE concentrations in the stems. Overall, the growth substrate strongly affected REE concentrations in leaves but not those in stems with a more strongly pronounced effect on LREE (*p* < 0.01) than on HREE (*p* = 0.05). Considering data from all mixed culture forms and P fertilizer treatments, leaf concentrations on substrate B were 64% (LREE) and 72% (HREE) higher (*p* < 0.05) than those on substrate A but with similar LREE/HREE ratio. Application of P fertilizer in monoculture significantly decreased LREE concentrations of leaves (by 48%) and LREE and HREE concentrations of stems both by 50% on substrate A, while on substrate B, this effect was not observable. Also, in the mixed cultures, there was no direct effect of P application and there were no differences in element concentrations between mixed cultured plants that received different fertilizers. Moreover, plants that received only 1.5 g m^−2^ P (NK) showed no differences in elemental composition between monocultures and mixed cultures. However, on substrate A, mixed cultures of barley with *L. angustifolius* that were treated with P fertilizer responded by a significant increase in concentrations of LREEs by 113% and HREE by 88% in leaves and 225% (LREE) and 200% (HREE), respectively, in stems compared to the monocultures.

**Table 6:** Concentrations (µg g^−1^ dw) of light rare earth elements (LREEs) and heavy rare earth elements (HREEs) and their ratio (LREEs relative to HREEs) in the plant parts depending on substrate (slightly alkaline substrate A and slightly acidic substrate B), P addition (NK: 1.5 g m^−2^ P; NPK: 3 g m^−2^ P) and culture form (monoculture: L0, mixed culture with 11% *L. albus* (Lal) and mixed culture with 11% *L. angustifolius* (Lan))

	Culture	Leaves			Stems		
		LREE, µg g^−1^ dw	HREE, µg g^−1^ dw	L/H, µg g^−1^ dw	LREE, µg g^−1^ dw	HREE, µg g^−1^ dw	L/H, µg g^−1^ dw
Substrate A							
Fertilizer							
NK	L0	0.44 ± 0.20A*B*	0.12 ± 0.09	4.4 ± 1.5a	0.08 ± 0.04(A)	0.04 ± 0.02(A)	2.5 ± 0.7
	Lan	0.41 ± 0.19	0.24 ± 0.21	2.7 ± 1.3b	0.04 ± 0.03(B)	0.15 ± 0.13	1.4 ± 0.9
NPK	L0	0.23 ± 0.06bB*B*	0.07 ± 0.02b*B*	3.7 ± 0.6	0.04 ± 0.02b(B)*B*	0.02 ± 0.01(b)(B)*B*	3.2 ± 0.2a
	Lan	0.49 ± 0.21a	0.12 ± 0.05a*B*	3.8 ± 0.7	0.13 ± 0.06a(A)*A*	0.06 ± 0.03(a)*A*	2.2 ± 0.3b*B*
	Lal	0.37 ± 0.15ab	0.10 ± 0.06ab	4.0 ± 0.9	0.07 ± 0.04ab	0.03 ± 0.02(ab)	3.2 ± 0.7a
Substrate B							
Fertilizer							
NK	L0	0.77 ± 0.28*A*	0.18 ± 0.07	4.2 ± 0.5A	0.09 ± 0.04	0.04 ± 0.03	3.1 ± 1.3
	Lan	0.58 ± 0.30	0.16 ± 0.09	4.4 ± 0.7	0.04 ± 0.01	0.02 ± 0.01	3.4 ± 0.6
NPK	L0	0.59 ± 0.14*A*	0.25 ± 0.18*A*	3.0 ± 1.2B	0.21 ± 0.19a*A*	0.13 ± 0.11(a)*A*	3.7 ± 2.6
	Lan	0.68 ± 0.31	0.21 ± 0.08*A*	4.1 ± 1.4	0.05 ± 0.01b*B*	0.012 ± 0.004(b)*B*	4.1 ± 1.4*A*
	Lal	0.48 ± 0.13	0.15 ± 0.07	3.4 ± 1.0	0.05 ± 0.01b	0.017 ± 0.007(b)	3.4 ± 1.0

Means ± SD (*n* = 5). Significant differences in yields and concentrations within a plant part and substrate were identified by MANOVA followed by Duncan’s post hoc test. Small letters show differences between means of monocultured and mixed cultured barley within a specific substrate and P treatment. Capital letters denote differences of concentrations in barley plants of a specific treatment between P treatments within a substrate. Capital letters in italics show differences of concentrations in barley plants between substrates at *α* = 5%

On substrate A, *L. albus* did not alter the mineral composition of the mixed cultured plants, irrespective of the P application. In contrast, on substrate B, NPK-treated mixed cultures with both *L. albus* and *L. angustifolius* significantly decreased the REE concentrations by a factor of 4 in the case of LREEs or even roughly 1 order of magnitude in the case of HREEs. It has to be noticed that these effects were only prevailing on slightly alkaline substrate A when plant stands of barley and mixed cultures of barley and *L. angustifolius* were treated with higher doses of P fertilizer.

### Accumulation of nutrients and REEs

**Fig. 1:**
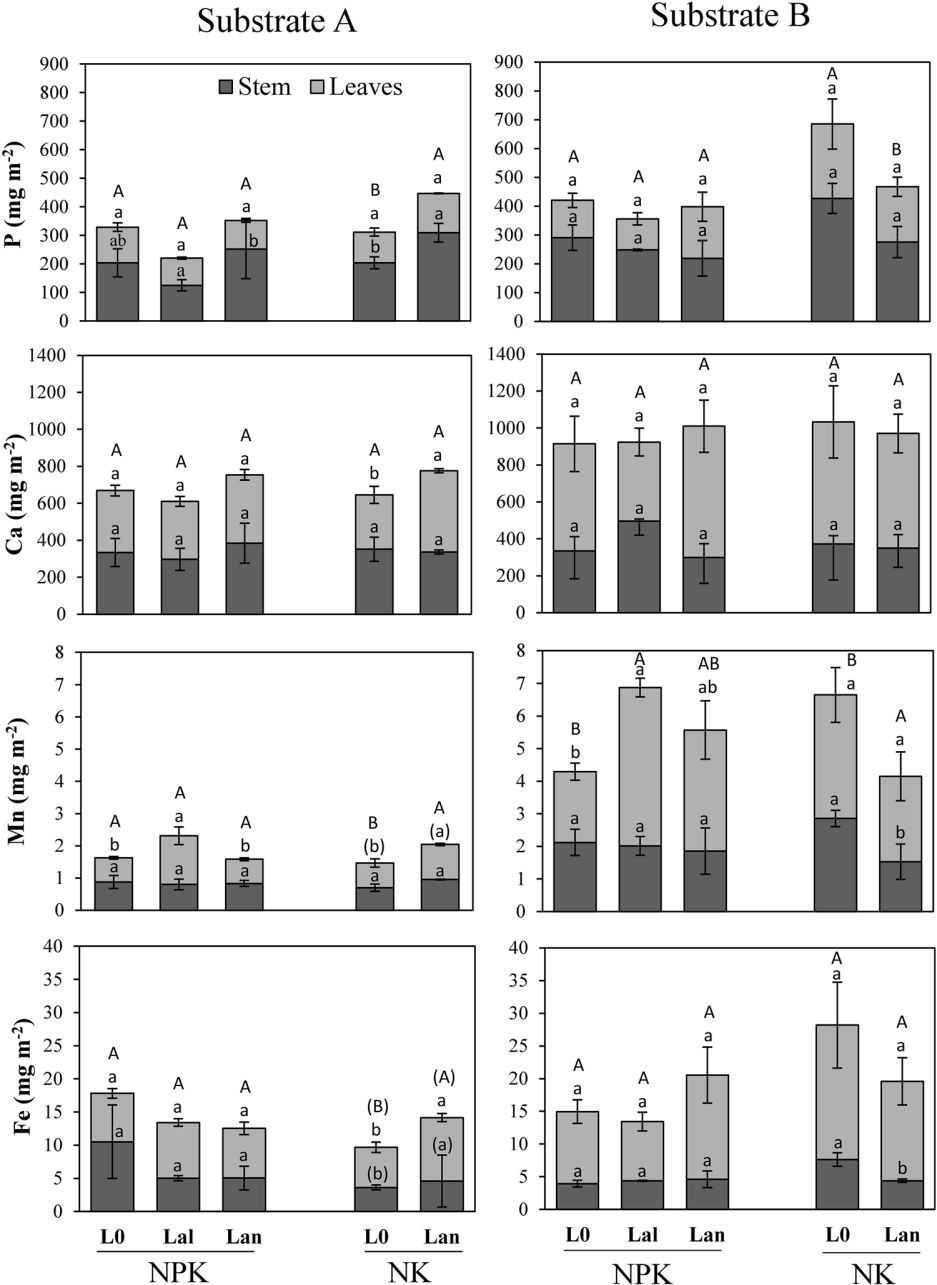
Total accumulation of nutrients in leaves, stems and shoots (total height of bars) of barley plants in monocultures (L0) and mixed cultures with *L. angustifolius* (Lan) or *L. albus* (Lal) on slightly alkaline substrate A and slightly acidic substrate B. On both substrates, the plants in different culture forms were treated with 3 g m^−2^ P (NPK) or 1.5 g m^−2^ P (NK). Means ± SD (*n* = 5). Differences among means were identified by MANOVA followed by Duncan’s post hoc test. Small letters denote differences in element contents within a specific plant part, substrate and P addition treatment. Capital letters show differences between shoot contents within the substrates and treatments at *α* = 5%

**Fig. 2:**
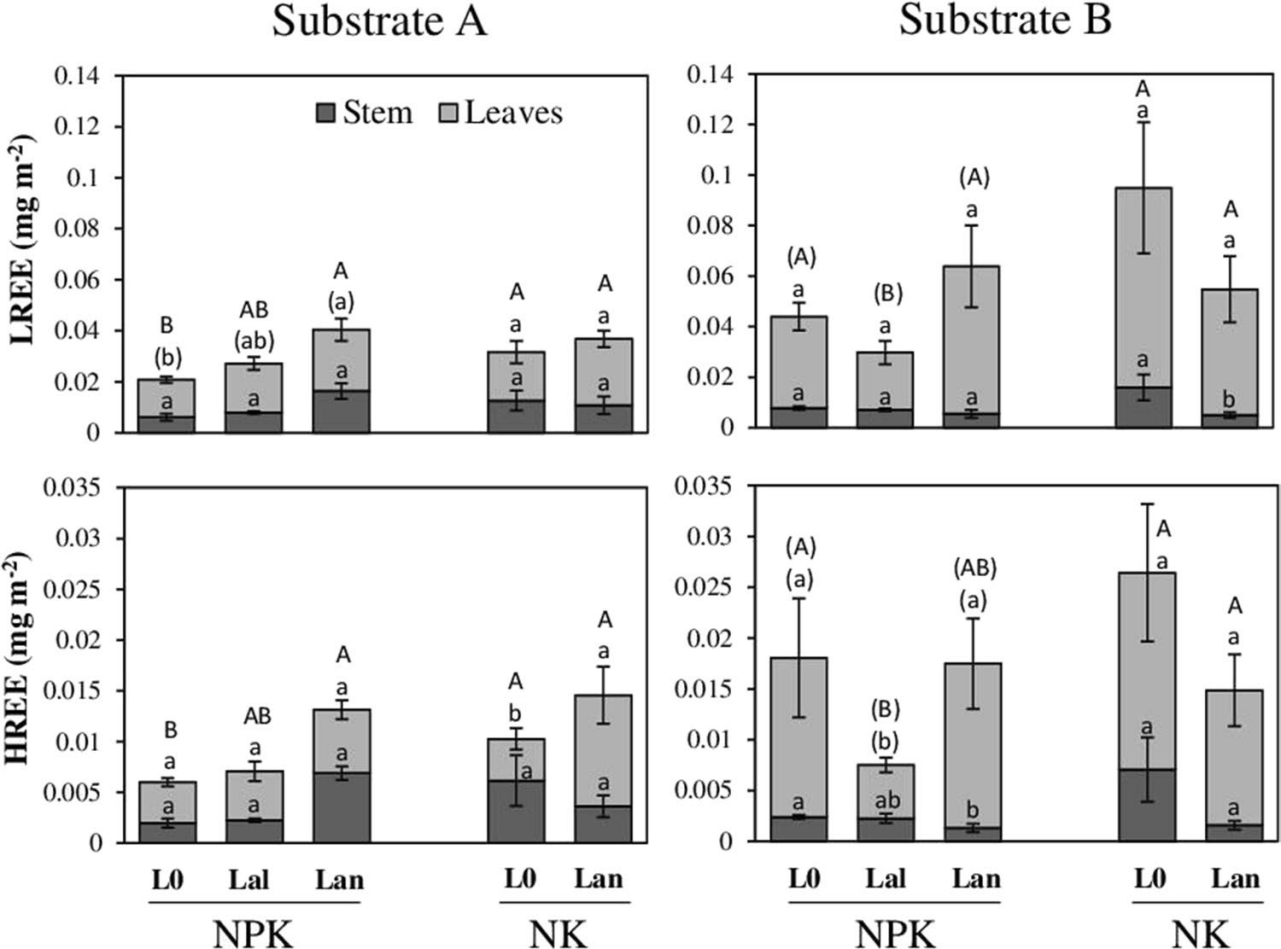
Total accumulation of nutrients in leaves, stems and shoots (total height of bars) of barley plants in monocultures (L0) and mixed cultures with *L. angustifolius* (Lan) or *L. albus* (Lal) on slightly alkaline substrate A and slightly acidic substrate B. On both substrates, the plants in different culture forms were treated with 3 g m^−2^ P (NPK) or 1.5 g m^−2^ P (NK). Means ± SD (*n* = 5). Differences among means were identified by MANOVA followed by Duncan’s post hoc test. Small letters denote differences in element constants within a specific plant part, substrate and P addition treatment. Capital letters show differences between shoot contents within the substrates and treatments at *α* = 5%

Considering the biomass of leaves and stems and the herein quantified element concentrations, amounts of elements in the respective plant tissues and whole shoot contents were calculated (Fig. 1 and Fig. 2). Plant leaves consistently contained significantly (*p* < 0.01) higher amounts of Ca (30%), Mn (44%) and Fe (87%) and especially of LREEs (265%) and HREEs (158%) than stems, except P which predominantly accumulated in plant stems with 78% higher amounts than in leaves. The growth substrate strongly influenced the element contents in leaves showing significantly higher amounts of all investigated elements in leaves of plants cultivated on substrate B compared to substrate A (Table 7). In stems, only contents of P and Mn were influenced by a general substrate effect (Table 7). Considering all P addition levels and culture forms, plants cultivated on substrate B contained 57% (P), 73% (Ca), 251% (Mn) and 97% (Fe) as well as 158% (LREEs) and 145% (HREEs) more of the investigated elements in the leaves. Additionally, the plants showed 43% (P) and 160% (Mn) more of the elements in stems on substrate B compared to substrate A without an effect from the substrate on Ca, Fe, LREE and HREEs in this plant tissue. Consequently, element contents in shoots that integrate results from both leaves and stems, respectively, were also affected by substrate showing higher contents of P (10%), Ca (18%), Mn (170%), Fe (23%) and LREEs (60%) and HREEs (13%) in shoots of plants that were cultivated on substrate B compared to plants on substrate A.

**Table 7:** Multifactor multivariate ANOVA based on leaf and stem contents (µg m^−2^) of barley plants, exploring for effects of the growth substrate, fertilizer addition (3 g m^−2^ P or 1.5 g m^−2^ P, respectively) and culture form (monocultures and mixed cultures)

Plant tissue	Source of variation	P	Ca	Mn	Fe	LREE	HREE
Leaves	Substrate	*	**	***	**	**	**
	Fertilizer	NS	NS	NS	NS	NS	NS
	Culture	NS	0.08	**	NS	(*)	*
	Substrate × culture	NS	NS	NS	NS	NS	(*)
	Fertilizer × culture	NS	NS	NS	NS	NS	NS
	Substrate × fertilizer × culture	(*)	NS	*	(*)	NS	NS
Stems	Substrate	(*)	NS	***	NS	NS	(*)
	Fertilizer	NS	NS	NS	NS	NS	NS
	Culture	NS	NS	NS	NS	NS	NS
	Substrate × culture	**	NS	(*)	NS	*	*
	Fertilizer × culture	NS	NS	NS	NS	(*)	NS
	Substrate × fertilizer × culture	NS	NS	NS	(*)	NS	NS
Shoots	Substrate	(*)	(*)	***	(*)	*	(*)
	Fertilizer	NS	NS	NS	NS	NS	NS
	Culture	NS	NS	NS	NS	NS	NS
	Substrate × culture	**	*	**	(*)	**	**
	Fertilizer × culture	NS	NS	NS	NS	NS	NS
	Substrate × fertilizer × culture	*	NS	*	*	NS	NS

*NS* not significant

(*)*p* < 0.1; *p < 0.05; **p < 0.01

The element contents in shoot biomass were not influenced by general effects of culture form and P fertilizer addition but rather depended on complex responses of different levels of plant tissue accumulation based on interactions of culture form and substrate properties as well as additional interaction effects of P fertilizer amendment (Table 7). Specifically, compared to *L. angustifolius*, intercropping with *L. albus* did not positively affect the accumulation of the investigated elements except that of Mn in leaves and shoots of barley plants on substrate B. On substrate B, the presence of *L. albus* increased Mn content in leaves by 116% and in shoots by 63% compared to monocultures, while on substrate A, *L. albus* increased the leaf Mn contents by 102% compared to monocultures. However, for LREEs and HREEs, *L. albus* significantly decreased the element contents in shoots (by 68% and 71%, respectively) and leaves (by 36% and 46%, respectively) when the plants grew on substrate B with 3 g m^−2^ P addition, while on substrate A, no effect of *L. albus* on REE accumulation in mixed cultured barley was observed.

Unfortunately, in this study, *L. albus* was solely cultivated on the two substrates with higher dosing of P fertilizer and, thus, further evaluations of responses of the mixed cultures to different P availabilities are not possible. However, considering mixed cultures with *L. angustifolius*, the effect of intercropping on element accumulation was strongly dependent on the growth substrate and P fertilizer addition. More specifically, on both substrates, there was no response of mixed cultured barley regarding the contents of P, Ca, Mn and Fe when barley and *L. angustifolius* were cultivated with a higher supply of P (NPK treatment). In contrast, when P supply was reduced (NK treatment) and barley was cultivated neighbouring to *L. angustifolius*, shoot contents of P, Mn and Fe increased on substrate A by 64% (P), 56% (Mn) and 62% (Fe). This was mostly caused by a significant increase in leaf contents, except for P, whereas on substrate B, the shoot contents of P, Mn and Fe decreased by 37% (P), 50% (Mn) and 37% (Fe), respectively, due to decreased accumulation in stems and leaves. Concomitantly, on substrate B, there were clear tendencies of a reduction of shoot LREE (by 44%) and HREE (by 46%) accumulation when plants were cultivated with *L. angustifolius* and 1.5 g m^−2^ P dosing compared to the monocultures. Under these conditions, *L. angustifolius* significantly reduced LREE contents in stems of barley by 69%. Also, on substrate B, the presence of *L. angustifolius* significantly reduced stem contents of HREEs by 46% in 3 g P m^−2^–dosed mixed cultures compared to the monocultures but without striking effects on bulk shoot contents which remained unchanged.

In contrast, on substrate A, mixed cultures with *L. angustifolius* significantly increased contents of LREEs (by 79%) and HREEs (by 96%) in shoots of barley compared to the monocultures. This can be attributed to a combination of increasing contents in leaves (60% increase for LREEs and 50% increase for HREEs) and in stems (169% increase for LREEs and 263% increase for HREEs) when 3 g m^−2^ P was given. For HREEs, this effect was also visible in leaves of plants that were treated with lower P doses (62% increase). However, the effect in leaves was not strong enough to influence bulk shoot contents of HREEs that remained unchanged compared to the monocultures. Due to a decrease in stem HREE contents, there was no effect on LREE plant stands treated with 1.5 g m^−2^ P.

### Phosphorus concentrations in lupin plants as affected by substrate and P supply

**Fig. 3:**
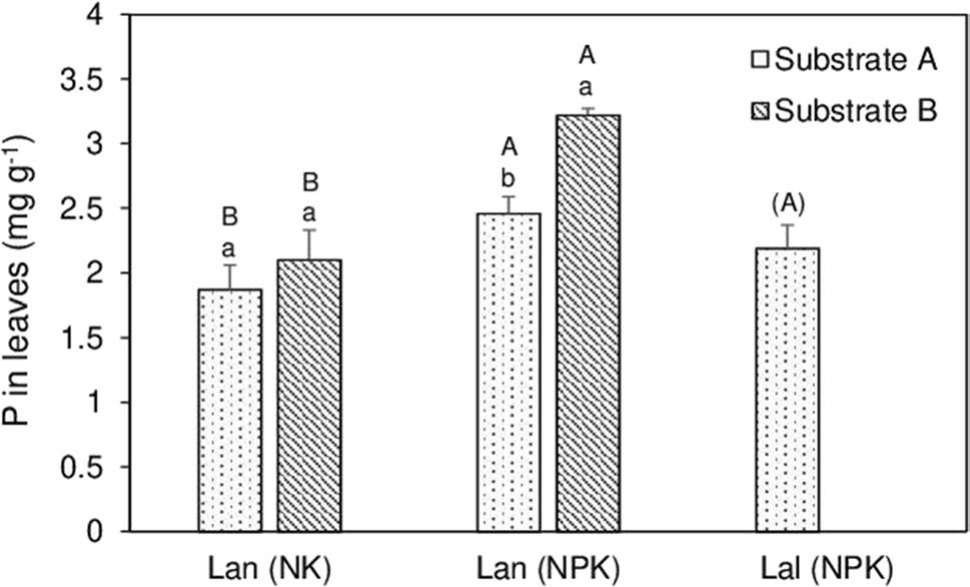
Leaf P concentrations in mixed cultured lupin plants (*L. angustifolius* (Lan), *L. albus* (Lal)) that received fertilizer with 1.5 g P m^−2^ (NK) or 3 g P m^−2^ (NPK), respectively. Means ± SD (*n* = 4). Significant differences among means were identified by *t* tests with Bonferroni adjustment. Small letters denote differences between the substrates within a certain P treatment. Capital letters show differences in P treatments within a specific substrate. Means with different letters are significantly different at *α* = 5%

Mixed cultures of barley and lupins that received only low doses of P (1.5 g P m^−2^) did not show significant differences in leaf P concentrations when plants cultivated on substrates A and B were compared (Fig. 3). Nevertheless, P concentrations in plants on substrate B were slightly higher (2.3 mg g^−1^) compared to lupins cultivated on substrate A (1.9 mg g^−1^). Generally, on both substrates, fertilization of the mixed cultures with P fertilizer significantly increased the concentrations of P and this effect was most visible on substrate B where NPK-treated plants reached up to 3.1 mg g^−1^ P in leaves. Here, plants of *L. angustifolius* displayed substantially higher P concentrations than plants on substrate A. *L. albus* was only cultivated under the NPK addition of substrate A, and thus, investigations of responses of the species to substrate and P supply were not possible. Compared to *L. angustifolius*, *L. albus* exhibited similar P concentrations when both species received NPK fertilizer (Fig. 3).

## Discussion

### Evaluation of carboxylate release in different lupin species

In the greenhouse experiment, exudation experiment was carried out as a means to evaluate the carboxylate release and, consequently, the nutrient acquisition efficiency of the cultivars of *L. albus* (Feodora) and *L. angustifolius* (Sonate) that were later used in the field experiment for intercropping with barley. Lupins are characterized by an extraordinarily high efficiency to mobilize sparingly available P, Fe and Mn in the rhizosphere through carboxylate release and acidification which is extensively documented in the literature (Cu et al. 2005; Lambers et al. 2013; Pearse et al. 2006; Wiche et al. 2016b), while barley is described as P-inefficient (Marschner 1995). The results successfully demonstrate that the response of the two species was divergent, showing a higher release of carboxylates in *L. albus* under P-deficient conditions, whereas *L. angustifolius* responded with a decreased release of carboxylates and the highest exudation rates under high P supply (Table 3). For *L. albus*, this is in congruency with the results from Pearse et al. (2006), Müller et al. (2015) and Neumann and Römheld (2000) who reported increased diffusion of citrate and malate as a consequence of metabolic shifts in carbohydrate allocation from shoot to roots in concert with increased biosynthesis of malate and citrate and decreased citrate turnover in the tricarboxylic acid cycle. Concomitantly, the decreased release of carboxylates in *L. angustifolius* suggests that this species (or the selected cultivar) lacks the ability to alter carboxylate metabolism following P deficiency similar to chickpea and *Brassica napus* (Pearse et al. 2006; Lambers 2022). Indeed, the total amounts of carboxylates released per plant were higher in *L. albus* whereas the exudation rates (per root dry weight) of both lupin species were similar under low P supply (Table 3). *Lupinus albus* is a cluster root-forming lupin species and generally produces more extensive root systems compared to *L. angustifolius* (Egle et al. 2003; Pearse et al. 2006; Clements et al. 1993). Since carboxylate release mainly concentrates on active cluster roots, the lower carboxylate release per unit root weight in *L. albus* observed in this study could be explained by a higher total root dry mass relative to the number of active root tip regions of *L. albus* in concert with no changes in biomass allocation under P-deficient conditions (Funayama‐Noguchi et al. 2015). This partly contradicts the previous findings of Pearse et al. (2006) who observed higher rates of carboxylate release per unit root mass in *L. albus* compared to *L. angustifolius*. We emphasize that the ability to respond to P deficiency varies substantially among different lupin species and even different genotypes within a species. More specifically, Egle et al. (2003) explored P supply–induced changes in malate and citrate release of different cultivars of *L. albus* and *L. angustifolius* and demonstrated a higher variation for *L. angustifolius* than for *L. albus*. The latter was characterized by a lower carboxylate release efficiency per unit root and responded to P deficiency with elevated carboxylate release, while all *L. angustifolius* cultivars showed the opposite response (Egle et al. 2003), which is in good agreement to our results. Here, adequately P-supplied *L. angustifolius* showed substantially higher carboxylate exudation rates and amounts of citrate released per plant individual compared to *L. albus* (Table 3). However, cultivar-dependent differences between our study and that of Pearse et al. (2006) cannot a priori be ruled out. Based on the above, it appears that *L. albus* should be preferably selected for intercropping aiming at improved plant nutrition in mixed culture systems, especially when plant growth is limited by P availability. On the other hand, the tested *L. angustifolius* cultivar seems to be suitable for improvement of nutrient supply on moderately fertile soils. With regard to a selection of lupin species, other substrate parameters, particularly soil pH, Ca and bicarbonate concentrations, are of additional relevance. Compared to *L. angustifolius*, *L. albus* is relatively tolerant against Ca and bicarbonate in soil solution and develops well on soils over a wide pH range from 5 to 8. However, in alkaline soils above a pH value of 7, iron deficiency can cause chlorosis (Duthion 1992). In contrast, *L. angustifolius* is calcifuge and high concentrations of bicarbonate may decrease root growth and increase carboxylate release, irrespective of the external P supply (Peiter et al. 2000).

### Effect of substrate properties on plant growth and nutrient availability to the plants

Considering the leaf nutrient concentrations which are commonly used as proxies for the nutritional state of plants (Hayes et al. 2014), it was obvious that on both substrates, the barley plants suffered from Mn and P deficiency indicated by leaf P concentrations close or even below to the critical value of 2 mg g^−1^ P and 50 µg g^−1^ Mn (Marschner 1995). The lowest concentrations of P and Mn (below 1.9 mg g^−1^ P and 20 µg g^−1^ Mn) were observed in plants on substrate A treated with 1 g P m^−2^ (Table 5). Surprisingly, comparing leaf, stem and shoot biomass on both substrates, we did not observe significant changes in plant yields between the substrates (Tables 4 and 5). Compared to substrate A, concentrations of P, Ca, Mn and Fe in barley leaves as well as bulk shoot contents (Fig. 1, Table 5) were significantly higher on substrate B, indicating an improved nutrient supply on this substrate with its slightly acidic pH. Furthermore, on substrate B, leaf P concentration of lupin plants was significantly higher than that on substrate A and significantly higher compared to *H. vulgare* (Table 5, Fig. 3), while on substrate A, leaf P concentration in unfertilized plants of *L. angustifolius* was similar to that of *H. vulgare*. Higher nutrient concentrations in lupins compared to *H. vulgare* can be explained by a higher nutrient acquisition efficiency of lupins (Pearse et al. 2006). Based on P concentrations determined by CAL extracts, both substrates were sufficiently supplied with P (Marschner 1995) but the phosphorus was most likely not present in plant-available forms. Substrate A was slightly alkaline (pH 7.9) (Table 1) which fosters the precipitation of sparingly soluble Ca phosphates (Mengel et al. 2001) and low solubility of Mn and Fe. In contrast, soil B was slightly acidic (pH 6.8) (Table 1) so that low specific sorption of P (Mengel et al. 2001) as well as higher solubility of Mn and Fe can be expected (Gupta and Chipman 1976). Generally, higher accumulation and concentrations of the nutrients on substrate B was not surprising (Fig. 1, Table 7). However, the higher availability of the elements on substrate B exhibited by higher tissue concentrations and shoot contents was not a priori predictable based on data of the sequential extraction where substrate A showed lower concentrations of P, Ca, Mn and Fe in mobile, exchangeable fractions (Table 2). On the contrary, substrate B was characterized by higher concentrations of P, Fe and Mn bound into organic matter and amorphous Fe oxyhydroxides (Table 2). This demonstrates that sequential extractions do not sufficiently describe the availability of elements since they do not integrate all soil-associated factors and plant-associated factors overlapping in the rhizosphere in time, space and function (Hinsinger et al. 2009; Vetterlein et al. 2020). This suggests that in this experiment, the higher availability of nutrients on substrate B rather depended on the mobility of the elements in the soil (once they are mobilized) as a consequence of pH and, thus, a lower reprecipitation/readsorption of mobilized elements in the rhizosphere of the plants than distribution of elements in operationally defined element fractions. In this light, we emphasize that CAL extracts (Table 1) exhibited a higher P availability on substrate B which was in agreement with the substrate-induced differences in tissue P concentrations in plants. This suggests that both the CAL–extractant solutions (acidified Ca lactate) and the plants were able to access moderately stable element pools through acidification and ligand–exchange reactions, especially the lupins with their efficient acquisition strategy.

### Relationships between the substrate, P fertilization and lupins on plant growth and nutrient availability in mixed cultures

In this experiment, we used a replacement model, where within the mixed cultures, barley was replaced with 11% of *L. albus* and *L. angustifolius* (Wiche et al. 2016a). Although there were slight reductions in yields following a replacement, growth substrate, different levels of P supply and intercropping did not affect plant yields of barley. With the exception of substrate A and on plots with 1.5 g P m^−2^ amendment, intercropping with *L. angustifolius* slightly increased the leaf biomass of barley (Table 5). Of course, plant growth and yield predominantly depend on the nutritional state of the plants which was experimentally controlled by substrate properties, the addition of P fertilizer and intercropping with P-efficient lupins (Lambers 2021). Moreover, the efficiency of intercropping strongly depends on the nutritional status of both the barley plants and the lupin plants because under conditions of increasing nutrient availability, the barley plants would cover their nutrient demands from soil resources and belowground traits of intercropping plants may not deliver additional benefits. Thus, positive effects of intercropping can be especially expected under conditions of moderate to low nutrient availability. However, as nutrient availability decreases, the root competition intensity between neighbouring plants increases (Schenk 2006; Craine and Dybzinski 2013). Especially in barley–lupin associations, the competing plant individuals are substantially different in morphological and functional traits above and below ground. As such, the resulting competition should be largely asymmetric with the lupins monopolizing soil P and micronutrient sources by exploiting the resource before the barley individuals are able to obtain it (Pearse et al. 2006; Schenk 2006). Consequently, nutrient facilitation in lupin–barley mixed cultures should especially occur in situations where barley is exposed to growth-limiting soil conditions. But, this should be where the lupins are still readily able to satisfy their own nutritional demands (Cu et al. 2005; Gunes and Inal 2009; Wiche et al. 2016b), or when other environmental stress factors and positive effects of barley for the lupins shift the balance between positive and negative interactions (Brooker et al. 2008). Unfortunately, we did not consider other soil resources and environmental factors in our study, and thus, based on our data, no further mechanistic interpretations are possible. In our experiment, the addition of the P fertilizer did not influence the P concentrations and contents of barley plants neither on substrate A nor on substrate B (Tables 4 and 5). Possibly, the differences in doses between the two treatments were not high enough (1.5 g m^−2^ or 3 g m^−2^ P) to generate a treatment-dependent difference in the plants’ nutrient supply. Furthermore, the barley plants did not export the P absorbed from roots to shoots (Schjørring and Jensén 1987). Increased P allocation to the grains (El Mazlouzi et al. 2020) influenced the P concentrations in vegetative plant organs, the leaves and stems, respectively. After 8 weeks of plant growth, barley already reached the reproductive stage. Also, based on the above, it is reasonable that the lupin plants strongly competed with barley for phosphate. In fact, the P concentrations in lupins significantly increased when P was added (Fig. 3), indicating a strong root competition for essential elements between lupins and barley. There is evidence that the importance of root competition increases relative to other factors with increasing resource availability in soil (Schenk 2006). Finally, resource facilitation in mixed cultures strongly depends on the nutrient status of the lupin plants, their responses through the release of carboxylates influencing the solubility of the elements in the rhizosphere and migration of elements between the intermingling root systems (Cu et al. 2005; Wiche et al. 2016a, 2017a). The availability of P and micronutrients was higher in substrate B than in substrate A (Table 1, Fig. 1). Therefore, the low performance of *L. angustifolius* and *L. albus* in mixed cultures with barley on substrate B (Fig. 1, Table 5) could be explained by the synergetic effects of reduced carboxylate release by the lupins, especially of *L. albus* (Table 3), and higher substrate-induced solubility of the elements fostering element uptake by the barley plants. Nevertheless, increased Mn concentrations and accumulation (Fig. 1, Table 5) in mixed cultured on substrate B indicate that cluster roots of *L. albus* were still active even when P fertilizer was added. It has to be noticed that even on substrate B, the plants were still undersupplied with Mn (Table 5, Section “Effect of substrate properties on plant growth and nutrient availability to the plants”) which is an additional factor triggering carboxylate release by lupins (Marschner and Römheld 1994; Lambers et al. 2013, 2015). Concomitantly, carboxylates of *L. albus* are known to strongly affect the availability of Mn as this species is considered a hyperaccumulator of Mn (Lambers et al. 2015). In this regard, lacking effects in mixed cultures with *L. angustifolius* might indicate a lower ability of *L. angustifolius* to respond to deficiency of Mn, while decreased accumulation of P and Mn in the presence of *L. angustifolius* could be due to the competition of barley and lupins for these nutrients.

On substrate A, intercropping with *L. angustifolius* slightly increased leaf P concentrations of low P–dosed plants above the critical level of 2 mg g^−1^, suggesting that the improved nutritional state of the barley plants was responsible for the increase in leaf biomass (Table 5). On this alkaline substrate, leaf and shoot nutrient concentrations and contents of barley were exclusively positively affected (Table 5, Fig. 1) on experimental plots with 1.5 g m^−2^ P addition although the leaf P concentrations of lupins suggested a lower P supply in *L. angustifolius* (Fig. 3) which should lead to decreased root activity of this lupin species (Table 3). However, in plots with a higher P supply, we observed a better plant growth of lupins (data not shown here) so that it is reasonable that the mobilized nutrients were initially taken up by the lupins without any positive effects on barley. Concomitantly, increased concentrations and accumulation of Ca, Mn and Fe in mixed cultures with lower P supply (Table 5, Fig. 1) most likely originated from resource facilitation under the growth-limiting conditions of substrate A, where neighbouring lupins improved the nutritional status of barley plants.

### Effect of substrates, P fertilization and lupins on the availability of REEs in mixed cultures

In soils, REEs share many chemical similarities with essential plant nutrients, especially calcium (Brioschi et al. 2013; Censi et al. 2014, 2017; Martinez et al. 2018; Wyttenbach et al. 1998). Thus, nutrient-bearing soil phases such as phosphates, organic matter and Fe oxyhydroxides are important hosts for these elements (Diatloff et al. 1993; Zhimang et al. 2000; Cao et al. 2001; Wiche and Heilmeier 2016; Wiche et al. 2016b). Accordingly, in the soil used for the field experiment, REEs were mostly present in fractions 3–5 and with slight enrichment in fraction 3 of substrate B (Table 2). Low soil pH and the presence of dissolved organic matter strongly impact the mobility and plant availability of REEs (Diatloff et al. 1993; Zhimang et al. 2000; Cao et al. 2001; Tyler and Olsson 2001; Pourret et al. 2007; Kovaříková et al. 2019). As such, the higher concentrations (Table 6) and accumulation (Fig. 2) of REEs on substrate B in comparison to substrate A can be attributed to a higher solubility of the elements in this soil. Higher accumulation of LREEs relative to HREEs observed in this study (Table 6, Fig. 2) closely follows the natural abundance of the elements in the substrates (Table 3). Furthermore, the literature indicates a preferential uptake of LREEs compared to HREEs (Censi et al. 2017; Martinez et al. 2018) due to the higher stability of HREE–organic complexes and stronger adsorption of HREEs at ion exchange sites in the soil. These, in turn, may have contributed to these results. Surprisingly, in this study, leaf concentrations of REEs were constantly higher than stem concentrations and the plants mostly responded by changes in leaf REE concentrations (Table 6). Although the literature indicates a clear trend of decreasing REE concentrations in the order roots > stems > leaves across many plant species and genera (Li et al. 2001; Wen et al. 2001; Xu et al. 2003; Tyler 2004; Brioschi et al. 2013; Yuan et al. 2018), some studies also reported a reversed concentration pattern showing higher concentrations in leaves than in stems, especially in cereals such as oat, wheat and rice (Wiche et al. 2016a, b; Kovaříková et al. 2019). Thus, different REE patterns among different plant species may reflect a species-specific mobility of REE within plants (Kovaříková et al. 2019) and our findings in barley support the described pattern for cereals.

Differences in substrates as well as intercropping with lupins impacted both leaf and bulk shoot contents of barley (Fig. 2), although in barley, the predominant portion of the shoot biomass consisted of stems (Table 5). Leaves only accounted for one-third of the total shoot biomass (Table 5), and changes in foliar REE absorption due to treatment measures were impactful enough to compensate the lower biomass of this plant part when total shoot contents are considered (Fig. 2). Similar to the findings for nutrients (see Section “Effect of substrate properties on plant growth and nutrient availability to the plants”), REE concentrations on substrate B were predominantly influenced by substrate without significant effects of P fertilizer addition or positive effects of lupins in mixed cultures. However, on substrate B, the presence of *L. albus* significantly decreased both shoot REE concentrations and contents, especially when the plants were fertilized with P which highlights an immobilization or uptake of the elements by the lupins under conditions where mobility of the elements is high. Unfortunately, our experimental design did not allow exploring the processes beyond these effects. Nevertheless, it is reasonable that the lupines with their extensive root systems and especially *L. albus* which produces more extensive root systems compared to *L. angustifolius* (Clements et al. 1993) did not only compete for essential elements such as P but also REEs. Although lupines are generally characterized by low shoot REE absorption so far (Wiche and Heilmeier 2016), their roots could represent important element sinks in soil where REEs are stored or adsorbed onto cell structures (Han et al. 2005), especially when root carboxylate release diminishes due to sufficient external P supply (Table 3).

On alkaline substrate A, the addition of P fertilizer significantly reduced both LREE and HREE concentrations in monocultured barley plants (Table 6). This can be attributed to a precipitation of the elements as hardly soluble REE phosphates at alkaline conditions (Saatz et al. 2016; Han 2020) or a “dilution” effect originating from slightly higher shoot biomass (Table 5) which is frequently reported for non-essential elements (Chien and Menon 1995). Compared to the monocultures, the presence of *L. angustifolius* significantly increased tissue concentrations and shoot contents of both LREEs and HREEs in mixed cultured barley. Increased REE availability in mixed cultures with lupins was already described by Wiche et al. (2016b) but without considering differences in substrates or nutrient availability. In the present study, positive effects of mixed cultures were only visible on the alkaline, P fertilizer–amended soil and in the presence of *L. angustifolius* which releases higher amounts of carboxylates under sufficient P supply (Table 3). Indeed, in view of the P-induced increase in carboxylate release observed in the greenhouse study (Table 3), these results were consistent with our previous findings (Wiche et al. 2016a, b); however, compared to *L. albus*, *L. angustifolius* is much less tolerant against high bicarbonate concentrations present at high soil pH as it can be expected in soil A (Peiter et al. 2000). High concentrations of bicarbonate can reduce the formation of lateral roots in *L. angustifolius* and may increase the carboxylate release in this calcifuge lupin species as it has been reported for lime-intolerant *Lupinus luteus*. However, Peiter et al. (2000) and Egle et al. (2003) reported a large variation in root responses among different *L. angustifolius* cultivars. In contrast, *L. albus* generally tolerates relatively high soil lime contents and does not respond to liming with reduced root growth and elevated carboxylate release (Peiter et al. 2000). Thus, the missing effects of *L. albus* on REE accumulation by barley can be widely explained by a reduced carboxylate exudation of *L. albus* due to sufficient P supply, while it seems reasonable that the significant effects of *L. angustifolius* are a consequence of carboxylates and protons released into the soil affected by high P supply and/or the bicarbonate in alkaline soil A. Most probably, under these conditions, the carboxylates released by lupins mobilized the REEs through the formation of soluble REE–carboxylate complexes (Wiche et al. 2017a) in the rhizosphere of the lupins. Since REEs are not essential for plant growth (Tyler 2004) and complexes of REEs are discriminated relative to their ionic forms during plant uptake (Han et al. 2005; Wiche et al. 2017a), the complexes were obviously not adsorbed by the lupins itself, enabling the movement to the intermingling barley roots where different chemical properties and microbial activity (Neumann and Römheld 2000; Renella et al. 2004) might have fostered the decay of complexes and thus root uptake and transport of REEs to the shoots of intercropped barley.

## Conclusion

We could demonstrate that soil-associated factors above plant-associated factors play a crucial role in determining REE fluxes in soil plant systems. Within a certain soil environment, application of 3 g P m^−2^ reduced the accumulation of REEs in barley monocultures, most likely through REE precipitation in the root zone. However, our results clearly show that P availability also indirectly affects REE fluxes in soil–plant systems by influencing the nutritional status of the plants, and thus, the chemical properties of the *meta*-rhizospheres of intermingling barley–lupin root systems. In barley–lupin associations, the mobilization of REEs in the rhizosphere of lupines and REE transport to neighbouring plants seems to depend on the species-specific ability to respond to different levels of P supply with carboxylate release. *L. angustifolius* cv. Sonate, a lupin cultivar that responds to increased P supply with increased carboxylate release, increased the accumulation of REEs in barley plants when the plants were additionally supplied with P fertilizer and cultivated on an alkaline soil characterized by low initial availability of REEs and nutrients. In contrast, on soil with high P and REE mobility, the presence of *L. albus* cv. Feodora, which responded to increased P supply with decreasing carboxylate release led to decreased REE contents in barley, most probably due to the root REE absorption of the lupins. Considering these factors, mixed culture cropping systems could be a powerful tool to enhance the accumulation of REEs in a sense of phytoremediation or phytomining on marginal soils, while at the same time, the mixed cultures with *L. albus* cv. Feodora could be deployed to attenuate REE accumulation in crop plants for food production, especially in REE-polluted soils. The processes involved in the results are not yet fully understood, and thus, elucidation of chemical element species in the rhizosphere of neighbouring plants and responses of different cultivars to P supply–induced REE mobilization remains a field of further research. Nevertheless, our findings suggest that interspecific root interactions involved in REE fluxes in legume–grass communities are influenced by species-specific strategies related to P acquisition and the nutritional status of neighbouring plant individuals.

## Supplementary Information

Below is the link to the electronic supplementary material.

Supplementary file1 (PDF 341 KB)

## Data Availability

Not applicable. Raw data (primary data obtained from HPLC or ICP-MS) has not been considered for publication in data repositories.
