# Supplementary material for: Effect of substrate properties and phosphorus supply on facilitating the uptake of rare earth elements (REE) in mixed culture cropping systems of Hordeum vulgare, Lupinus albus and Lupinus angustifolius
Source: Environ Sci Pollut Res Int. 2022 Mar 28;29(38):57172–89. doi: 10.1007/s11356-022-19775-x (PMC9395493; doi:10.1007/s11356-022-19775-x)
Supplement: Supplementary file 1 — Supplementary file1 (PDF 341 KB) [file 11356_2022_19775_MOESM1_ESM.pdf]

Effect of substrate properties and phosphorus supply on facilitating the uptake of rare earth elements (REE) in mixed culture cropping systems of *Hordeum vulgare*, *Lupinus albus* and *Lupinus angustifolius*

Environmental Science and Pollution Research

Nthati Monei [ab#], Michael Hitch [ac], Juliane Heim[d], Olivier Pourret[e], Hermann Heilmeier[b], Oliver Wiche[b#]

[#]the authors contributed equally to this manuscript

[a]Tallinn University of Technology, Institute of Geology, Tallinn Estonia
[b]Technische Universität Bergakademie Freiberg, Institute of Biosciences, Biology/Ecology Group, Freiberg, Germany
[c]Curtin University, Western Australian School of Mines, Bentley, Australia
[d]Technische Universität Bergakademie Freiberg, Institute of Biosciences, Environmental Microbiology Group, Freiberg, Germany
[e]UniLaSalle, AGHYLE, Beauvais, France

Corresponding author: Oliver Wiche (Oliver.Wiche@ioez.tu-freiberg.de)

Substrate A					Substrate B				
A-H-NK-Lan11-1	A-H-NPK-Lal-11-1	A-H-NPK-Lan11-3	A-H-NK-5	A-H-NPK-Lan11-5	B-H-NPK-1	B-H-NPK-Lan11-5	B-H-NPK-5	B-H-NPK-Lan11-4	B-H-NK-Lan11-5
A-H-NPK-1	A-H-NK-2	A-H-NPK-Lal11-4	A-H-NK-Lan11-3	A-H-NPK-Lan11-2	B-H-NK-Lan11-1	B-H-NK-3	B-H-NPK-Lal11-4	B-H-NK-Lan11-3	B-H-NPK-4
A-H-NPK-Lal11-2	A-H-NK-Lan11-5	A-H-NPK-3	A-H-NPK-Lal11-3	A-H-NPK-2	B-H-NPK-Lal11-1	B-H-NPK-3	B-H-NPK-2	B-H-NK-2	B-H-NPK-Lan11-2
A-H-NK-3	A-H-NK-Lan11-2	A-H-NK-1	A-H-NK-4	A-H-NPK-Lan11-1	B-H-NK-Lan11-2	B-H-NK-Lan11-4	B-H-NPK-Lal11-3	B-H-NPK-Lan11-1	B-H-NK-5
A-H-NPK-Lan11-4	A-H-NPK-4	A-H-NPK-Lal11-5	A-H-NK-Lan11-4	A-H-NPK-5	B-H-NPK-Lal11-2	B-H-NK-4	B-H-NK-1	B-H-NPK-Lan11-3	B-H-NPK-Lal11-5

Decription of the plotcode:
A/B = substrate type
H = *Hordeum vulgare*
NPK/NK = fertilizer application (NPK: 3 g P m^{-2} ; NK: 1.5 g P m^{-2})
Lan11/Lal11 = 11% *Lupinus angustifolius*/11% *Lupinus albus* (*H. vulgare* monoculture if not indicated)
1-5 = replicates

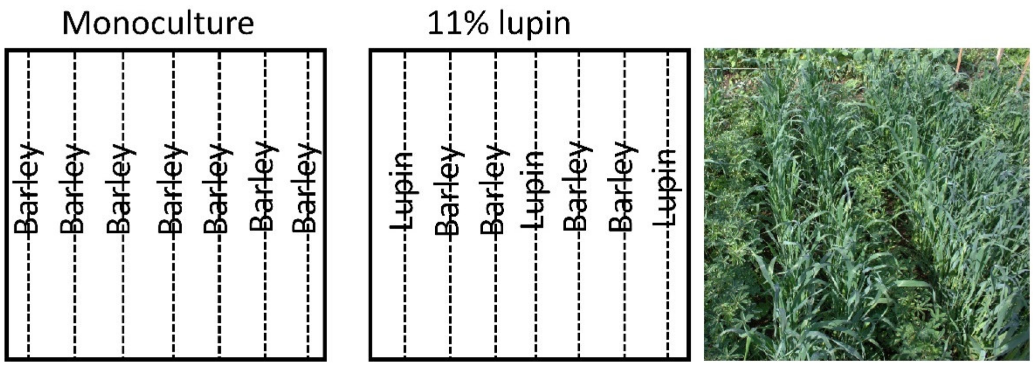

Online Resource 1: Experimental setup (above) of the field experiment with the experimental plots of *H. vulgare* monoculture and mixed cultures of *H. vulgare* with 11% *L. albus* (Lal) or 11% *L. angustifolius* (Lan) on two different substrates (A, B) with two different P-doses (1.5 g P m^{-2} (NK) or 3 g P m^{-2} (NPK)). The figure below shows the planting pattern in monocultures and mixed cultures.
